# Extensor Tendon Dislocation at the Metacarpophalangeal Joint of Both Ring Fingers Caused by a Specific Hand Posture in a Shiatsu Therapist

**DOI:** 10.1155/2020/6842986

**Published:** 2020-09-22

**Authors:** Mariko Kamiya, Gen Sasaki, Kensuke Ikuta, Hideaki Miyamoto, Michio Kimura, Hirotaka Kawano

**Affiliations:** Department of Orthopaedic Surgery, Teikyo University School of Medicine, 2-11-1 Kaga, Itabashi-ku, Tokyo 173-8606, Japan

## Abstract

A 43-year-old female shiatsu therapist complained of sudden snapping of the metacarpophalangeal joints (MCPjs) of both ring fingers during a specific hand posture. The extensor tendon of the ring finger was dislocated ulnarly when the MCPj of the ring finger was flexed and deviated ulnarly and the MCPj of the middle finger was extended. Surgical exploration revealed an attenuated radial sagittal band. We plicated the juncturae tendinum of the extensor digitorum communis between the middle and ring fingers and released the ulnar sagittal band partially to centralise the extensor tendon excursion. Twenty-six months postoperatively, the patient regained full active and passive range of motion of all fingers without extensor tendon dislocation or snapping in either hand during work.

## 1. Introduction

Extensor tendon dislocation at the metacarpophalangeal joint (MCPj) of the hand has four etiologies: congenital origin, degenerative, traumatic, or spontaneous sagittal band rupture [1–3]. Degenerative dislocation of the middle finger extensor tendon, which is the most frequent etiology, usually occurs in patients with rheumatoid arthritis [3]. We report a rare case of spontaneous dislocation of the extensor tendons of both ring fingers caused by a specific hand posture in a shiatsu therapist.

## 2. Case Presentation

**Figure 1:**
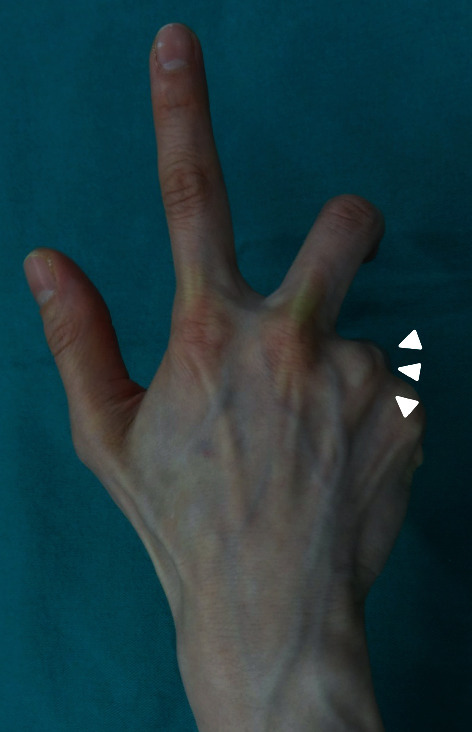
(a) Shiatsu-specific hand posture. (b) When the metacarpophalangeal joint (MCPj) of the ring finger is flexed and deviated ulnarly with concurrent extension of the MCPj of the middle finger, the extensor tendon of the ring finger dislocates ulnarly (white arrowheads).

A 43-year-old female shiatsu therapist with 20 years of experience complained of sudden snapping of the MCPj of the left ring finger (her nondominant hand) and pain in the dorsum of the MCPj. The patient did not have any conditions that would predispose her to sagittal band laxity including prior trauma to the hand. Two weeks later, the snapping and pain occurred in the right ring finger. On physical examination, the extensor tendons at the dorsum of the MCPjs of both ring fingers dislocated with a specific hand posture (Figure 1). On actively extending the MCPjs of the ring fingers, the extensor tendons reduced with snapping. However, the ring finger extensor tendon did not dislocate if both the middle and ring finger MCPjs were flexed together. Radiography revealed no MCPj deformity. The patient did not comply with nonoperative treatment such as MCPj extension bracing because she could not continue work with bracing.

**Figure 2:**
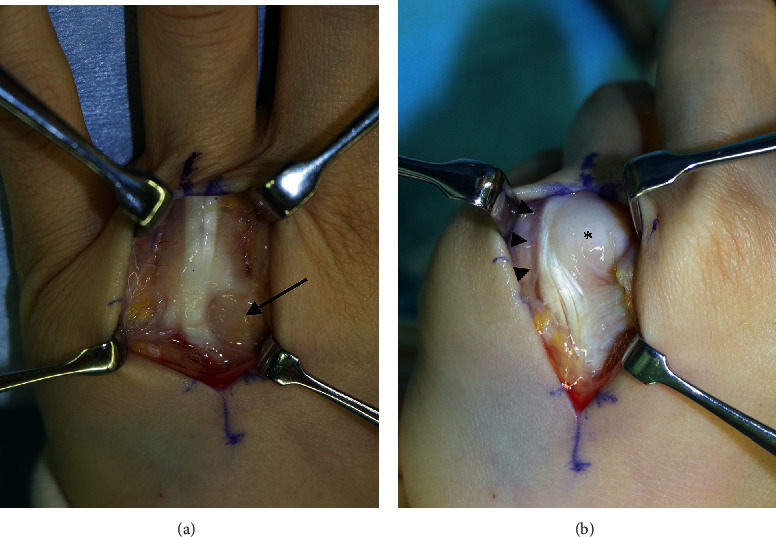
Surgical image for the extensor tendon of the ring finger of the left hand. (a) Attenuated radial sagittal band (black arrow). (b) The metacarpal head of the ring finger pops through the attenuated sagittal band (asterisk) with the extensor tendon dislocated ulnarly (black arrowheads) in the shiatsu-specific hand posture.

Four weeks after the first visit, we performed reconstruction of the extensor tendons of the both ring fingers with wrist blocks. Surgical exploration through a longitudinal curved skin incision at the dorsum of the MCPj of the ring finger revealed a markedly attenuated radial sagittal band and ulnarly dislocated extensor tendons bilaterally (Figure 2). We plicated the juncturae tendinum of the extensor digitorum communis between the middle and ring fingers and released the ulnar sagittal band partially to centralise the extensor tendon excursion without dislocation, through the full range of motion of the MCPj intraoperatively. We also tested the range of motion of the active finger.

Postoperatively, we applied a bulky hand dressing with a forearm-based volar plaster splint to the MCPj in the extended position in order to prevent inadvertent passive flexion of the MCPj. Active flexion and extension exercises were performed from 1 to 6 weeks postoperatively. Six weeks postoperatively, we removed the splint and allowed all MCPj movements. Six months postoperatively, the patient resumed work as a shiatsu therapist without extensor tendon dislocation and snapping at the MCPjs. All finger movements were performed with no limitations in either hand. Twenty-six months postoperatively, the patient remained pain-free during work without extensor tendon dislocation or snapping in either hand.

## 3. Discussion

Dislocation of the extensor tendon usually presents with symptoms of pain and snapping at the MCPj during MCPj flexion. In our case, however, the extensor tendon was dislocated only when the MCPj of the middle finger was extended and the MCPj of the ring finger was flexed with ulnar deviation. In acute injuries, conservative treatment may provide satisfactory results [4]. When nonsurgical treatment fails to correct the subluxation, surgical treatment may be required. Reconstructive techniques include methods using an extensor digitorum communis tendon slip, juncturae tendinum, lumbrical tendon, and palmaris longus with a suture anchor [5]. Because chronic or spontaneous cases are associated with degenerative insufficiency of the injured sagittal band, various reconstructive techniques have been described using a variety of graft sources and attachment sites [6, 7]. Reconstructive techniques using grafts are associated with a number of potential problems such as donor site morbidity and dysfunction at the MCP joint because of ineffective graft pathways or attachment sites. In chronic cases, a concern might be raised that the ruptured sagittal band will be atrophied and difficult to use. On the other hand, some authors report satisfactory results with only reefing and imbrication of the extensor hood in these chronic cases [8, 9]. Although prior reconstructive techniques have reported satisfactory results, this technique is simpler than those reconstructive techniques and may offer the additional advantage of not substantially altering adjacent anatomy.

The sagittal band is the primary stabilizer of the extensor tendon at MCPj, keeping the tendon in position during flexion and extension. Failure of the sagittal band to centralise the tendon, which may be the result of trauma, laxity, or congenital absence, causes dislocation of the extensor tendon [10]. Usually, the dislocation occurs on the ulnar side of the middle finger because the radial component of the sagittal band is more prone to injury than the ulnar component. In addition, the metacarpal head of the middle finger is more prominent, and the attachment of its extensor tendon to the transverse fibers, compared to those of the three other fingers, is loosen [11, 12].

**Figure 3:**
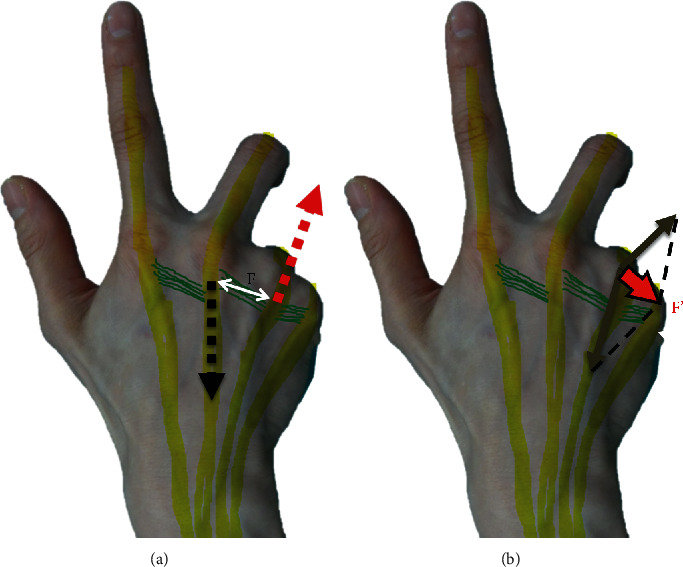
Schematic images of an ulnarward translation force to the extensor tendon of the ring finger. (a) Force to the sagittal band between the middle and ring fingers. In the middle finger, when the metacarpophalangeal joint (MCPj) was extended, the extensor tendon was retracted proximally (black arrow). However, in the ring finger, when the MCPj was flexed, the extensor tendon was retracted distally (red arrow). The traction force to the sagittal band and the juncturae tendinum between the middle and ring fingers (F) may become larger and result in sagittal band and juncturae tendinum attenuation. (b) Force-vector combination to the extensor tendon at the MCPj of the ring finger resulting from MCPj flexion and ulnar deviation may develop an ulnarward translation force (F′).

Contrary to most reports, our case indicated that excessive traction stress by repetitive use of a specific posture may contribute to rupture of the radial component of the sagittal band of the ring finger (Figure 3). This rare case of spontaneous tendon dislocation at the MCPj of the ring finger highlights that shiatsu-specific hand posture is a possible cause of sagittal band tear, in which the MCPj of the middle finger is extended and the MCPj of the ring finger is flexed.
